# Trying to make race science the “civil” science: charisma in the race and intelligence debates

**DOI:** 10.1007/s11186-022-09481-5

**Published:** 2022-04-26

**Authors:** Kushan Dasgupta, Aaron Panofsky, Nicole Iturriaga

**Affiliations:** 1 Institute for Society and Genetics, University of California, Los Angeles, CA, USA; 2 Department of Criminology, Law and Society, University of California, Irvine, CA, USA

**Keywords:** Charisma, Scientific authority, Public sphere, Race, Intelligence

## Abstract

When studying science contexts, scholars typically position charismatic authority as an adjunct or something that provides a meaning-laden boost to rational authority. In this paper, we re-theorize these relationships. We re-center charismatic authority as an interpretive resource that allows scientists and onlookers to recast a professional conflict in terms of a public drama. In this mode, both professionals and lay enthusiasts portray involvement in the scientific process as a story of suppression and persecution, in which only a few remarkable figures can withstand scrutiny and take on challengers with dignity. Description and elaboration of these figures and the folklore surrounding them sets in motion the interpretive processes by which some actors become charismatic leaders and others charismatic followers within science, ultimately providing alternative symbolic resources for an embattled research agenda to accrue legitimacy. To illustrate, we use the case of Arthur Jensen – a deceased intelligence researcher and the intellectual father to contemporary texts like The Bell Curve – and the circles of hero worship that admirers inside and outside academia have created to praise him. Using this perspective to study Jensen and his admirers demonstrates how the perennial race and intelligence debates gain a kind of symbolic power, unrelated to their scientific merit or racist appeal, which enables such debates to thrive and persist in the public sphere. More generally, our approach identifies contemporary processes by which scientific ideas can gain public authority even when their intellectual merit has been deemed dubious.

What is the relationship between charismatic and rationalized authority in science? To the degree that scholars have investigated this dynamic, they typically position charisma as a kind of meaning-laden stopgap, providing affective and symbolic resources to augment the managerial processes enabled by workflows, protocols, or standardized deliberation (Gustin, 1973; Lorimer, 2007; Thorpe & Shapin, 2000). This reading is also consonant with studies of other technocratic environments, like commercial corporations or government bureaucracies (Bear, 2013; Choi & Mody, 2009; Khurana, 2002). However, scholars of religion and modern politics have cautioned against this formulation. Though this reading pushes past some of the synoptic and commonsensical approaches to charisma, which describe charismatic and rationalized authority as inimical to one another, scholars argue that it “diffuse[s] Weber’s model completely” (Lainer-Vos & Parigi, 2014:477). By linking charisma to a general need for meaning, it “effectively saps the concept of its ability to grasp ‘unusual’ situations” (Reed, 2013:260), when failures or other charged social situations become cast in Manichean terms, excitement legitimates obedience to an extraordinary individual, and success becomes redefined in terms of the ability to execute achievements or miracles.

In this article, we build on these concerns, developing an account that re-theorizes the significance of charisma for science. As we demonstrate, charisma has the potential to be more than a meaningful tool in the managerial aspects of science, and instead offers interpretive resources that allow scientists and onlookers to recast a professional conflict in terms of a public drama, redefining the stakes, players, and boundaries of struggle. We develop this account with an empirical case study related to the history of race and intelligence research (Tucker, 1996), a stream of academic scholarship which positions intelligence (i.e. I.Q.) as something primarily hereditary and something racial groups possess varying capacities for. Perhaps most well-known through books like *The Bell Curve*, this area of research is a broader phenomenon and ongoing feature within academia, often given life by efforts within psychology, behavioral genetics, neuroscience, and other disciplines. By drawing on this case, our article also utilizes insights regarding charisma to comment on this form of racialized, biodeterminist reasoning and rethink reasons it possesses enduring appeal.

Specifically, we focus on a puzzling feature within the history of this research occurring among academic researchers, pundits, and non-academic hobbyists: their considerable admiration for Arthur Jensen. Jensen is a deceased psychologist and intelligence researcher, one of the high-profile scholars whose writings and public engagement in a previous era helped popularize race and intelligence research (Panofsky, 2014; Tucker, 1996). He is most well-known for publishing a 1969 article in the *Harvard Educational Review*, in which he aggregated evidence to claim that genetic differences between racial groups explain a significant part of racial differences in IQ scores. What we draw attention to in this article is less about the individual efforts of Jensen and more about the collective efforts of some colleagues, readers, and onlookers, who have created an admiration society or circle of hero worship, one which in earlier eras existed in print publications and today lives on as well through social media and other internet-based venues.

For instance, users on Twitter re-post the words of honor that fellow psychologists have bestowed upon Jensen, like the following: “He was irreplaceable in the sense that you often learn more from a thinker who challenges opinions you tend to take for granted^1^.” Admirers also create memes featuring images of Jensen studiously reading a book, captioned with quasi-provocative quotes of his: “I will be ashamed the day I feel I should knuckle under to social-political pressures about issues and research I think are important for the advance of scientific knowledge^2^.” This only scratches the surface as others go on treasure hunts, tracking down and reposting digitized recordings of Jensen’s television appearances^3^, or write lengthy blog entries, social media posts, or Twitter threads, all extolling praise for Jensen.

There are at least three reasons as to why this veneration for Jensen is fairly puzzling and informs our turn to charisma theorizing. First, in terms of scholarship, Jensen’s methods and findings are outdated. Contemporary racial hereditarians profess that their positions come from simply following the science^4^, and even when they concede that it is not settled how genes determine race and intelligence, they claim that future advances will eventually settle this debate. However, Jensen’s entire oeuvre consists of psychometric research or other kinds of research conducted prior to the advent of genomics, which in today’s world provides the means for adjudicating whether science is “settling the debate” on specific matters of heredity and behavior^5^.

This leads to the second peculiarity, which is that as a psychometrician, Jensen could only speculate about the biological etiology that would actually underlie the heritability of IQ scores. On multiple occasions, he even agreed it would be impossible to move beyond speculation^6^. For example, in different publications he explained that to move past speculation would require breeding experiments that treated members of racial groups like Mendel treated varieties of peas – something that he admitted would be impossible.

Third, in the absence of clear, research- or evidence-based rationales, one might turn to the social context that informs racial hereditarians’ worldviews. Race and intelligence research often draws controversy (Panofsky, 2018), and therefore one could argue that racial hereditarians praise Jensen because he “took a stand” against public pressure to not discuss genetics in relation to intelligence. In this explanation, there is a leader-follower dynamic that sustains this admiration. While this argument begins to go in the right direction, it is limited in its explanatory power. This is because Jensen was not the first to take such a stand nor was he alone among his contemporaries (Segerstråle, 2001). Furthermore, there has been no shortage of people willing to stake similar positions since his career ended (Evans, 2014). The idea that Jensen is a leader because he was a confident figurehead only pushes the question further: why do racial hereditarians single Jensen out for such admiration when others have taken similar positions and received similar pressures?

We argue that in order to understand this phenomenon we must analyze Jensen as someone who has not just become a leader among racial hereditarians but rather as someone who has specifically become a charismatic leader (Joosse, 2017; Reed, 2013; Weber, 1978). This explains why his colleagues and onlookers position him as a unique and extraordinary exemplar among other racial hereditarians – “a king among men,” as the title of a special issue devoted to Jensen in a leading intelligence journal declares. As we will detail later, his extraordinariness comes not only from standing against social pressures, but from how others perceive him as embodying a scientific asceticism that is hard to maintain in the face of such pressures and controversies. The efforts of present-day racial hereditarians to deify Jensen on the internet are in part the work that a charismatic community performs to sustain a leader’s legacy after death as well as an extension of the work that others have been doing for decades to elevate Jensen.

Our analysis will reveal two important points about charisma within science. First, charismatic reverence provides an interpretive prism that structures and organizes claimsmaking. In the case of racial hereditarians, by professing admiration for Jensen, speakers identify that what makes him special – which is his ability to weather public backlash – is also evidence of the wider public’s intolerance. Therefore, charismatic reverence provides not simply an affective boost for technocratized environments, but rather a mode or set of scripts for articulating criticisms about incivility and close-mindedness in science. Second, charismatic reverence provides an organizational prism that crosscuts the existing structure of affiliations, disciplines, universities, research institutions, or media organizations. In the case of racial hereditarians, it does so by providing an alternative source of legitimacy – a way out, so to speak – as racial hereditarianism lives in a state of embattlement rather than validation among science professionals. As adherents channel emotional energy stemming from grievances about so-called suppression of research and redirect it towards admiration for a central figure, both an intellectual grouping and a charismatic community form, creating a public that spans various institutionalized environments and that can play host to this public drama about science gone amok.

Finally, on a broader note, studying the role of charisma provides new insights about the intellectual movements for race science. Most analyses of race and intelligence research, and biodeterminist accounts of race broadly, emphasize scientific or political dimensions, namely how these forms of science violate scientific consensus or are scientific misappropriations in service of racist, political ends (Evans, 2014; Fischer et al., 1996; Saini, 2020; Tucker, 1996). In either interpretation, racial hereditarianism is analyzed as a form of reasoning that appeals to adherents. Charisma illustrates that there may be another dimension to this phenomenon. Adherents may be invested in this line of thinking because it also provides emotional and other interpretive resources to deal with grievances, all of which involves a role in a charismatic community as the acolyte and defender of a scientist-cum-free speech martyr (Joosse, 2017). In this regard, racial hereditarianism is not just an ideological or misguided intellectual project, but it is also a relational phenomenon, and the preservation of those relationships have their own logic. For opponents of biodeterminism, it is quite possible that neither appeals to evidence nor “going to battle” with racial hereditarians are likely to be sufficient responses, as both will invigorate the sense of persecution and excitement upon which a charismatic community thrives.

This article is divided as follows. First, we review relevant literatures, particularly those that touch on the question of how influence accrues within science, how charisma plays a role within a science, and how previous scholars have analyzed race and intelligence controversies. Next, we introduce Jensen’s case, beginning with an exploration of the professional and political conditions that enable charismatic authority to become salient and operative within science, as well as identification of the rhetorical moves that bring charismatic authority into fruition. Following this, we dig deeper into the charismatic network that arose to venerate Jensen, exploring the various organizational and interpretive affordances that charisma provides to embattled scientists. Finally, we end with a discussion of how charisma re-shapes our understanding of science-public relations and how a public drama approach to charisma provides new ways of studying issues related to meaning in science.

## Science and legitimacy: beyond professional validation

Charisma typically does not factor into ideas about influence and leadership in the sociology of science. Instead, scholars usually position professional validation as central to understanding how scientists accrue influence and gain steam for a research agenda (Collins, 1983; Merton, 1942; Oreskes, 2019). Professional validation consists of the procedural practices – e.g. academic training, peer review – that constitute a discipline and its intellectual craft (Fox, 1994; Shils, 1997; Starr, 1982). Some conceptualize these practices as forms of boundary-drawing, by which scientists demarcate good from bad or better from worse forms of science (Gieryn, 1983). Others see these practices as having a related but also inverse function, in which scientists exhibit generosity, or the willingness to signal competency to another and bring him or her into the professional fold (Eyal, 2013). Regardless of the perspective, professional validation of a procedural sort is seen as central to the mix of factors by which scientists gain legitimacy and the capacity to be influential to fellow peers and the wider public (Eyal & Levy, 2013). While this framework reflects much of scientific practice, it in many ways underappreciates an important caveat within the Weberian imagination from which it originates: many sources of legitimacy actually intermix in much of social and professional life (Thorpe & Shapin, 2000:549; Weber, 1978:216). For instance, several recent studies illustrate how traditional authority – “the sanctity of age-old rules,” “personal loyalty which results from common upbringing” (Weber, 1978:226–27) – animates the sensemaking practices of scientists. Hamann (2016) describes how obituaries are attentive to academic “ancestral lines,” in which they identify which scientist was a doctoral student of a particular noteworthy figure or describe who worked in the stead of Planck and Einstein. Similarly, Hermanowicz’s (1998, 2016) studies of academic scientists identify a cadre of professionals who describe themselves as more interested in the qualities that come with collegiality, comradery, and stewardship rather than advancement in a scientific profession. Though these examples fall short of linking these alternate sources of legitimacy to the question of influence, they demonstrate that such sources continue to have staying power.

We posit another reason for adjusting our understandings of how legitimacy and influence accrue: for some, the process of professional validation and those tasked with its handling may become the targets of disillusionment or ire (Hilgartner, 1997). In such instances, other forms of legitimacy may not just live alongside professional validation but rather be positioned as a substitution. This predicament may emerge for a number of reasons, but it is not uncommon for members of any profession to feel that requirements are stacked against them or that senior figures and authorities are no longer impartial (Kempner et al., 2011). As others suggest, this sentiment may be heightened when felt within science professions, as the conflicts that give rise to such disillusionment may seem to contradict the common notion that scientists strive first and foremost for truth and objectivity (Moore, 2006).

## Scientific charisma: from managerial guidance to public drama

Such situations, in which standards or mores seem to be unsettled, are often the context within which charismatic authority emerges and gains legitimacy (Reed, 2013). In its basic formulation, the concept of charisma explains that figures who seem inhumanly extraordinary can become the vessel for affective investment or devotion among those who are aggrieved and desire transcendent guidance about complications in the present (Reed, 2013; Weber, 1978). Such devotional ties can become the basis for leadership if the charismatic individual sustains achievements – or miracles, in the most classical formulation – that prolong followers’ sense that the leader is exemplary.

While there is a tendency in both scholarship and commonplace wisdom to treat the affective properties of charisma as inimical to the calculative modes of rational authority (Dow, 1978; Katz, 1972), the notion that institutions might co-habit both is no longer a new idea (Lainer-Vos & Parigi, 2014). Though this insight is more inchoate in science studies, traces of it do exist, mostly demonstrating charisma’s role as an affective or meaning-laden tool in project management efforts. Thorpe and Shapin’s (2000) analysis of how Oppenheimer ran the Manhattan Project provides probably the clearest illustration. Collaborators recall that against security concerns which emphasized compartmentalization, Oppenheimer facilitated the free flow of information by playing the role of “a good host with his guests.” In other cases, studies bring this insight to the opposite circumstance; in the absence of task compartmentalization, charismatic research directors serve as a managerial substitute, by using face-to-face encouragement to create collaboration that reduces redundancies (Lengwiler, 2006). In sum, when scholars investigate how charisma and scientific rational authority interact, they typically present charisma as something that gooses along decision-making in specialized contexts, by providing affective or symbolic resources for a manager to skillfully execute a less-than-perfect, rationalized workflow.

We focus on a different kind of problem within science for which scientists might turn to charismatic guidance – how embattled scientists strive to cultivate legitimacy (Craciun, 2016). While some embattled scientists might resign themselves to some state of internal banishment and grumble about their discipline (Kempner et al., 2011; Martin, 1997), others like those in this article’s case may actively fight back. Some responses may aim to re-calibrate a discipline so that it may include their marginalized research (Hess, 2004), but other responses may have different aims, such as reputation preservation (Craciun, 2016) or the creation of dissident subcultures (Hess, 1993; Locke, 2009). These kinds of responses offer scientists the opportunity to make “character work,” a necessary part of the construction of charismatic authority, a dedicated aspect of their public claimsmaking (Jasper et al., 2018; Joosse, 2012). As such, we conceptualize the cultivation of charismatic authority not so much as an intended response by embattled scientists to their grievances – the way religious charismatics sometimes originate (Barnes, 1978) – but more as a modality that emerges as embattled scientists pursue other problem-solving approaches.

Studying charisma in this manner helps reconnect the concept’s role in science back to at least three of the original aims in Weber’s formulation. First, as many point out, Weber largely described charisma as a “breakthrough” phenomenon (Parsons, 1963:xxxii), connected to “extraordinary or revolutionary situations or moments” (Reed, 2013:260). While operational uncertainty or knowledge ambiguity involved in scientific discovery can be unsettling, these features reflect a kind of routine unsettlement that by and large minimizes the scope of charismatic domination. As Weber (1978:1134) himself wrote: “Every event transcending the routines of everyday life releases charismatic forces…which are subsequently weakened again by everyday life. In normal times the powers of the village chief are very limited, amounting to little more than arbitration and representation.” Altogether, the extant literature on science and charisma has produced a specific kind of analysis, focused on “the way in which elements of charisma get incorporated into well-institutionalized patterns of action in an established or (relatively) stable social system” (Reed, 2013:260). This certainly is an important phenomenon worthy of treatment, given that Weber dedicated a section of *Economy and Society* to the routinization of charisma. However, it is a form of analysis that effectively gives short-shrift to the hallmark properties of the theory in its application to science contexts.

This brings us to the second point, which, as Reed (2013:256) puts it, is that charisma has “a broadly political application,” in that it relates to dynamics pertaining to domination, obedience, and the issuing of orders. This helps us re-conceptualize the scope of charisma’s application in science, by identifying the types of situations that might constitute “revolutionary” or “extraordinary” moments. In this regard, charisma is not just a tool to smooth over scientific or epistemic uncertainty, but it is also an interpretive prism with which leaders and followers organize and enact a public drama about some kind of conflict within science, such as allegations of suppression, perceived mistreatment of colleagues, or the willingness to submit to professional consensus, to name a few.

Third, charisma involves a “definite social structure” (Joosse, 2017:337; Weber, 1978:1119). Referred to as the “charismatic community” or the “charismatic aristocracy” in Weber’s original writings, this structure involves followers whose participation is “formally voluntary” and “a dictate of conscience.” In his original writings, Weber (1978:1116) signaled that that such relational dynamics could take root in science, as he wrote that the “imagination” of the German mathematician Karl Weierstrass was the same as any “artist, prophet – or demagogue”: one that evinces a sense the leader has been “seized” by his work, “revolutionizes [others] ‘from within,’” and impels an “inner subjection to the unprecedented and absolutely unique.”

In this regard, charisma creates a form of togetherhood that is at the very least autonomous and orthogonal to the prevailing organizational forms or structures of affiliation within science. This involves followers who hope “to share in the social, political or religious esteem and honor in which [the charismatic leader] is held” rather than search for “compensation, titles or ranks” (Weber, 1978:1119). Thus, subordinates do not leave interactions or communications with the charismatic leader simply more affectively invigorated to bring scientific or technological developments into fruition or more invested in the hierarchical forms of leadership that accompany research and development. Rather, they also leave invested in what Weber terms “a communal relationship,” which makes veneration of the leader a meaningful aim in and of itself. Ultimately, charisma creates within the institution of science a particular relational project, as actors set in motion scripts and forms of interaction which aim to make normative and multiply the ranks based on such veneration.

## Studying race science and racial hereditarianism in dramaturgical terms

Academics and others interested in race science or racial hereditarianism exist in a liminal state of acceptance vis-à-vis their peers (Panofsky, 2014, 2018). While their research interests are often far from the mainstream, these players continue to publish in academic journals – albeit, sometimes more obscure or less cited ones. And while their research often receives scorn, their ability to do such research has also been vigorously defended in public statements by other academics. This contested state of acceptance has meant that a perennial controversy has accompanied questions about race, biology, and behavioral attributes like intelligence. While this could be the material of high drama, with few exceptions (Gieryn and Figert, 1986) scholars have yet to analyze these episodes in such terms.

Instead, when scholars have previously asked “why do these debates persist” or “why do these ideas gain support,” the predominant response has focused on scientific or ideological rationales: that scientists either truly believe these ideas despite the questionable evidence presented, or that such ideas provide support for certain sociopolitical aims that these scientists privately hold (Flynn, 1980; Lewontin, 1995; Rose et al., 1984). Along these lines, a sizable literature has developed, challenging the intellectual merits of these ideas or uncovering collaborations among right-wing ideologues, white supremacist organizations, and intelligence researchers (e.g. Fischer et al., 1996; Tucker, 1996).

While race and intelligence research may be scientifically convincing or ideologically expedient to some, we make the case that such aspects of the story only partially explain what contributes to the debate’s longevity. We argue that events in the course of this debate’s history have enabled participants and onlookers to re-interpret it in terms of a different political conflict, involving the loss of civility and open-mindedness. Some scholars directly involved in these controversies, while speaking mostly to the scientific merit of this debate, have provided drips of commentary in which they highlight this possibility. For instance, Jerry Hirsch, a psychologist and behavior geneticist who debated with Jensen in academic journals, drew attention to these dynamics in an issue of *Educational Theory*. While lamenting about “inarticulate” responses to Jensen, Hirsch (1975:6) wrote:

Time and again the opposition to Jensenism has resorted to inarticulate and self-defeating hooliganism, so easily perceived as fascist interference with academic freedom and unfettered scientific inquiry. Their negative accomplishment has often been to stimulate newspaper stories and editorials extolling the courage of the Jensenists in their fearless pursuit of “knowledge.”

Thus, even one of Jensen’s sharpest critics noticed that there was a symbolic terrain to this debate which cultivated support for Jensen separate from any science argument. If responses could not be coordinated and written properly, interlocutors could be characterized in negative terms and storylines about “courage” and “fearlessness” could emerge. Another critic, neuroscientist Steven Rose, made a similar observation, stating that student protests against Jensen had turned him into a “martyr” in the press (Rose et al., 1973).

Altogether, these quotes from Jensen’s contemporary interlocutors suggest an account worth considering: that the interpretation that Jensen is an unfairly persecuted figure provides a kind of legitimacy that allows his ideas to see another day in the sun, regardless of the scientific merit or ideological valence of his ideas. This legitimacy, however efficacious it may be, would be unable to stand on its own if it were simply a matter of principle and belief. Like others, it needs to be generated and buttressed, and that’s where the study of charismatic authority comes into play.

Before we describe the cultivation of charismatic authority in this story, one aspect of charisma theorizing needs to be clarified in relation to Jensen. The story that we tell begins before Jensen’s entrance into race and intelligence research and continues after his death. Based on some understandings of charisma, death may complicate the analysis (Reed, 2013:283–84; Weber, 1978:246–49). If charisma is one of the routes to establishing legitimate domination, what happens when the individual for whom the obsequious fall in line exits the world? To understand this dynamic, we draw from recent perspectives on charisma written in the last decade, which take a decisively relational approach to advance the concept (Joosse, 2012, 2017; Reed, 2013). We begin by recognizing that in sociological terms the challenge that death represents is, as Reed (2013:284) puts it, the loss of “[an individual’s] timing and ability to master interpretations that center on themselves as the central causal agent in the universe.” Therefore, in theory, leaders can retain their “magic” if others carry on the orchestration of such interpretations, an arrangement which others have convincingly demonstrated can occur in their studies of “follower side” dynamics in charismatic communities (Joosse, 2017:336). Given the perennial nature of the race and intelligence debate and grievances about suppression in academia, it is not altogether surprising that the charismatic community formed in relation to Jensen seems to be continuing past his death. This is the story that we tell next.

## The construction of charismatic reverence

### Forging the mantle of charisma: professional and political conditions

We begin by elucidating the features that allow charismatic authority to become a dynamic factor in science. As recent writings which emphasize the processual and relational dimensions of charisma illustrate (Joosse, 2017; Reed, 2013), certain conditions, beyond personality attributes, have to be in place to afford others the grounds for perceiving such individuals as extraordinary and worthy of elevation. In particular, the beginning of Jensen’s arc demonstrates two salient factors: a broad professional conflict that positions one cadre of scientific practitioners as marginal, and the entrance of a player with seemingly legitimate standing in the field who takes up claimsmaking on behalf of the marginal position. Together, these create the conditions for the player to be described as “not another crank” – or in the case of Jensen, “not another race scientist” – laying the interpretive groundwork for a story about scientific martyrdom to emerge.

Two noteworthy predecessors help illustrate the history of this conflict and the conditions within it that primed a charismatic mantle for Jensen to don. Jensen was not the first figure in the post-war years, when a new scientific consensus emerged that treated race as a non-biological concept, to attempt to resurrect race science (Tucker, 1996). One cadre of scientists, whom psychologist and historian William Tucker (1996:151) terms the “segregationist scientists,” took on a race-focused agenda shortly after the Brown v. Board of Education decision. Among them was Henry E. Garrett, a past president of the American Psychological Association. Throughout the early 1960s, these scientists produced little original research and instead produced a litany of products summarizing previous research to service segregation policy. To keep up with the pace of desegregation efforts, they sought any publication venue and partnered with non-scientific public spokespersons who claimed to possess scientific knowledge. Together, these tactics seemed to erode the veneer of dispassion that they may have claimed to hold as scientists, a problem further compounded by their overt segregationist politics.

The second noteworthy party is William Shockley, recipient of the 1956 Nobel Prize in Physics for inventing the transistor, who in the early 1960s turned to studying the question of supposed “dysgenic” trends in humanity (Shurkin, 2006). On the one hand, Shockley used his scientific know-how to advance race differences research in a more measured manner compared to the segregationist scientists, emphasizing what was presently known in research and framing his goal as simply wanting to make “heredity-environment uncertainty” with regards to race differences a matter of exploration (Hirsch, 1981). On the other hand, understanding that his Nobel Prize celebrity status helped him garner attention, Shockley also employed provocative claimsmaking in an effort to continue to accrue publicity (Pearson, 1992). Among these efforts, for instance, was a piece outlining a list of thought experiments, one of which was a program that would reward people with sub-100 IQ to be sterilized and administered by bounty hunters.

In short, what both the segregationist scientists and Shockley demonstrate is that race differences research in the post-war period prior to Jensen’s arrival was populated by crude hereditarians (Panofsky, 2014:76). Whatever expertise these actors – and other contemporaries of theirs – possessed, they employed it in a way that may have yielded some success but damaged their credibility. For anyone interested in such research, it was difficult to argue with the position that the most public personas involved seemed to be manipulating science or conducting themselves in ways that warranted rebuke.

These factors laid the ground for Jensen’s public reception. Upon finishing his graduate work in 1956, Jensen was for the initial decade of his career a rather conventional psychologist teaching in the Graduate School of Education at University of California-Berkeley^7^. He researched learning differences among children, and though he had conducted a post-doctorate fellowship with Hans Eysenck, a well-known contributor to the hereditarian position, much of his initial research concluded that “cultural disadvantages” or “environmental conditions” accounted for most differences^8^. These circumstances would slowly begin to change in 1966 and 1967, during a fellowship at the Center for Advanced Study in the Behavioral Sciences (CASBS) at Stanford University. During this time, Jensen began work on a book manuscript about intelligence and heritability and struck up a friendship with Shockley, who as a physics professor at Stanford delivered a lecture to CASBS fellows about “IQ-heredity-environment-uncertainty” (Jensen, 1992; Tucker, 2007). According to Jensen, this fellowship year precipitated his studious foray into the role of genes in intelligence differences.

In 1969, he published “How Much Can We Boost IQ and Scholastic Achievement?” – his 100+ page article in the *Harvard Educational Review* (*HER*) – which drew intense interest among academics and non-academics alike and for which he would most be associated with for the remainder of his career. Similar to his predecessors, Jensen employed rather basic research techniques – literature review and aggregating existing studies – to make his point, but he qualified his use of these techniques in some important ways, given the prior history of the conflict (Panofsky, 2014:75–76). Like Shockley, Jensen framed his inquiry in terms of explaining what existing research said and what conclusions could be drawn, in contrast to the segregationists’ posture that existing research definitely proved racial inferiority. However, unlike Shockley, he refrained – at least initially – from using his approach to excoriate fellow scientists for ignoring genetics. Instead, he presented his approach as a sober assessment of a policy-relevant question: “how much can we boost IQ and scholastic achievement?” Additionally, he used previous genetics research to assert not that the environment was irrelevant, but rather that aside from severe malnutrition or poor neonatal and infant care, for most individuals the environmental conditions contributing to intelligence were satisfied. Finally, he couched the subject of race differences in a small but important part of the paper rather than make it the entire focus. These features make up only a portion of the 100+ page *HER* paper, but they illustrate how Jensen adopted a different posture compared to previous colleagues who entered into race differences research: one that more convincingly sustained the impression that such research was simply an application of professional activity rather than commitment to crude hereditarianism.

This impression of professionalism was further compounded by the sense that when the *HER* article was published, Jensen burst onto the scene, so to speak. He had not used his research to comment on segregation during the cycles of landmark civil rights policymaking years earlier. Thus, pundit Joseph Alsop (1969b) declared:

“A leading American educationist has now said what none has dared to say before…It would be nice to denounce these statements as mere ugly racist bosh. But one cannot do this with the formidably buttressed paper published in the Harvard Educational Review by Dr. Arthur Jensen…”

In other words, Jensen’s stance was seen more convincingly as a matter of scientific conversion rather than commitment to dogma: going from a conventional educational and developmental psychologist, who previously gave deference to cultural disadvantages or the environment, to one who had become thoroughly convinced by the weight of evidence that genetics was the dominant factor^9^.

For these reasons, after the publication of the *HER* article, Jensen became a spokesperson par excellence of the hereditarian viewpoint among supporters and even skeptics^10^. Observers positioned Jensen’s piece as the proper contribution that the hereditarian side of the race and intelligence debate had been needing; Alsop (1969c) declared that “this is, in fact, a paper that needs to be taken very seriously.” Daniel Patrick Moynihan reportedly told contemporaries that “the winds of Jensenism are blowing through Washington with gale force,” even though the segregationist scientists had made political in-roads (Cohen, 1974). In short, Jensen had achieved a kind of credibility that had heretofore escaped hereditarian researchers.

This credibility afforded Jensen a number of resources. On the one hand, he gained a reputation as a technically gifted scientist – “it seems unlikely that Dr. Jensen’s data can be challenged,” Alsop (1969a) declared in one of his many editorials about the subject (see also Horn, 1974; Sowell, 1973). But he also earned a kind of legitimacy with which admirers and observers could inoculate him from charges of extremism. Conservative commentator William F. Buckley, Jr. (1969) cautioned that Jensen should not receive the same treatment that often befall the “ideologists of racism” who take such findings and “mount campaigns of I-told-you-soism with truly ugly implications.”

When it became apparent that the public was conflicted over the implications of the *HER* article, Jensen and other racial hereditarians took two paths, mirroring the kinds of resources available to them. In one, they toiled away in back-and-forths with progressive scientists in academic journals, debating the merits of their data and the interpretations that could possibly be drawn. In another, they sought to delegitimize the criticisms and protests coming their way by building on the characterization of reasonableness that had been bestowed upon Jensen. When invited to respond to “environmentalists” about intelligence, Jensen often prefaced or concluded his responses with statements like the following:

“The presently small handful of dissenters who argue that genetic factors play no part in IQ differences are not unlike the few persons living today who claim the earth is flat” (Jensen, 1978).

“For scientists who research and write on the nature of human intelligence but do not uphold the current equalitarian dogma that differences in mental abilities are wholly or largely due to inequalities in the school and social environments, the traditional ‘ivory tower’ of academe turns into a beleaguered bastion. The attacks come as much, or more, from inside as outside” (Jensen, 1973).

It’s through this second path of defense – appealing to the characterization that Jensen is reasonable – that Jensen emerges as a martyred figure, as we detail in the next section.

In brief, Jensen’s episode brings into relief some basic conditions within science that create the basis for charismatic authority. Importantly, we see how central a professional conflict is to the genesis of charisma, creating the conditions for a research agenda to live in a state of embattlement rather than validation. As the history of the segregationist scientists and Shockley demonstrates, this state of embattlement in and of itself would not be enough for contributors to be interpreted as martyrs. However, it provides the political context for a seemingly legitimate, professionalized player’s treatment within a debate to be characterized as unwarranted and unreasonable, enabling such a player’s performance and temperament to become as meaningful an object of public discussion as his/her research.

### Embellishment: resignifying the terms of debate, from embattled scientist to renunciate or from politics to “truth”

What our analysis is beginning to reveal is that charismatic reverence provides an interpretive framework for re-narrating political conflict within science. When the proper background conditions are in place, embattled practitioners get to tell a story about science gone amok, in which the establishment’s unfair administration of scrutiny, critique, and attack against a seemingly legitimate player becomes a focal point. As this section will further flesh out, tales about the persecuted player’s ability to withstand such treatment become central to this process of contesting science. Not only do they provide the grounds for illustrating and reconveying who is on the side of right and wrong, but they also resignify the political nature of the debate – by portraying the beleaguered player’s willingness to face denunciation time and time again as a sign that a research agenda is on the side of truth, and the establishment’s excoriation as a sign that social or political considerations are censoring scientific inquiry.

Such viewpoints had become commonplace shortly after the publication of the *HER* article. When news began to break that student groups at universities were protesting Jensen’s speaking engagements, the New York Times ran an editorial column^11^ titled “Campus Totalitarians,” decrying “the subjugation of science” and describing such behavior as “the mark of Fascist, Nazi and Stalinist revolutions.” To be sure, some of Jensen’s critics responded in ways that provided material for this interpretation. For instance, in one back-and-forth, prominent biologist Richard Lewontin, one of Jensen’s most vocal critics, described the events surrounding “Jensenism” as similar to those surrounding “Jansenism,” the 17^th^ century Catholic movement that emphasized predestination and original sin, and against which the Catholic Church issued declarations of heresy and a papal condemnation (Lewontin, 1970). In this manner, interlocutors contributed to the sense that Jensen’s new career arc, as a figurehead and martyr, was constituted through engagement with “colossal players” – legitimate actors, or “sparring partners who are capable of testing the mettle that should befit anyone claiming ‘superhuman, or exceptional powers or qualities’” (Joosse, 2018:934). For instance, in his review of Jensen’s first book written after the *HER* article, psychologist John Horn (1974:546) pronounced:

Heros are made, not born, and something similar can be said of martyrs and other assorted defenders of a faith…[Jensen] has responded, as one might expect a martyr to respond, with a rather pained, detached, and controlled expression of righteousness.

In short, tales about Jensen’s performance and character had become a resource for interpreting this public controversy involving science, providing evidence that something detached, transcendent, and exceptional must be at stake.

However, a closer examination of the tales that fellow psychologists, other hereditarian scientists, and lay admirers tell about Arthur Jensen indicate a quizzical but ultimately telling feature. At a superficial level, the tales suggest that Jensen lacks charisma in the commonsense understanding of the word. At the same time that they recall memories of Jensen and build him up, numerous colleagues of his point out that he notably lacked demonstration of emotion. “The friends…know immediately that emotionality is no important part of Art,” recalls colleague Helmuth Nyborg (2003:xvi) in the opening chapter to a festschrift edited in Jensen’s honor. Elsewhere, Charles Murray, co-author of *The Bell Curve*, states that part of the reason Jensen built up the polarizing reputation that he did was because he was “obtuse,” lacking the ability to read situations properly (Woo, 2012a).

These qualities, however, are part of the same set of features that ultimately makes Jensen exceptional and heroic to his colleagues and admirers. As people describe Jensen, they emphasize that these oddities pertaining to presentation of self are what also made him unbothered by and blasé about the controversies mounting around him. For instance, one admiring colleague alleges that Jensen had “a touch of Asperger’s syndrome^12^.” Upon offering that armchair diagnosis, he adds:

“He was absolutely unflappable in the face of enormous hostility and criticism. He just stuck to trying to explain to people what the data were. No matter the data might be he felt he could explain it in a neutral way.”

In other words, to observers, Jensen’s inability to perceive social cues ground what seem to be an otherwise peculiar quality – a willingness to step into one battle after another with his detractors and offend audiences along the way.

As others see it, Jensen had a quality lacking among contemporary scientists. He was willing to face incendiary situations and take on public beratement in the name of research in a way that very few are willing. Nyborg (2003:xv) explains in similar terms why he thinks Jensen’s career is worth remembering: “it reveals the remarkably fine personal qualities and the rare application of Gandhian principles of an eminent scientist that stood headstrong and almost alone in a true Ibsen’s sense against a dreadfully strong head wind.”

Importantly, as Nyborg’s commentary begins to illustrate, many of these acolytes and admirers qualify that what makes Jensen even more noteworthy is that his public engagement came from some source of inspiration as much as scientific commitment. As they recount it, he had little interest in arguing for the sake of arguing, getting even with adversaries, or, even worse, capitalizing upon the fame that came from controversy. Rosalind Arden (2003:553), another contributor to Nyborg’s festschrift, puts it in this manner: “Arthur is a renunciate. He has chosen the stony path of scientific truth over the smoother course of popularity and public acceptance.”

In summary, stories about a figure’s willingness to duel with the scientific establishment provide the next step for bringing charismatic authority into fruition. Not only do they underscore the science establishment’s irresponsibility, but they allow embattled practitioners to reinterpret the state of beleaguerment that accompanies a marginalized research position.

As we see here, Jensen’s ability to withstand criticism – especially its vociferous forms –becomes proof that opposition to the establishment is principled rather than political. Thus, praising his miraculous abilities provides a new interpretive lens for supporting an embattled research position, one that draws attention to and finds validation with his performance rather than his research per se. Ultimately, by conjuring such accounts, a motley collection of academics and admirers had turned themselves into a charismatic community with Jensen at the center – a feature that we flesh out more thoroughly next.

## Recognition and devotional comportment: or, how reverence transmits charismatic authority across time and space

### Part 1: the importance of practice

Once charismatic authority is in play, acolytes actively work to sustain engagement and conviviality based on the hero worship that stories about martyrdom entail. This is because, as Joosse puts it, “the coin of the realm within all charismatic communities is charismatic attention from the leader, a form of attention that is elicited through the devotional comportment of adherents” (Joosse, 2018:181). In other words, keeping this interpretive approach to embattlement alive becomes its own relatively autonomous activity, as it involves forms of social currency – i.e., charismatic attention and devotional comportment – that are at the least orthogonal to or in tension with the conventional standards of achievement in scientific activity.

Academics’ and lay admirers’ investment in charismatic community and conviviality is highly palpable in the ways they signal their veneration of Arthur Jensen, which sometimes seem like condensed and caricatured versions of Weber’s writings on charisma. For instance, Jensen’s acolytes frequently compare him to Gandhi (Arden, 2003; Miele, 2002; Nyborg, 2003). Elsewhere, one colleague invokes “Caesar” to describe Jensen’s career arc (Stanley, 1987). The payoff to these disproportionate characterizations becomes evident in one Twitter thread, in which some proceed to compare Jensen to arguably the most famous charismatic figure in all history. The thread, initiated by a philosopher of science critical of his discipline’s treatment of hereditarianism, begins with a screenshot of an email the philosopher received more than one decade earlier from Arthur Jensen, who writes^13^:

Your book on heritability is absolutely excellent! I wish it had been available some 25 or 30 years ago; it might have spared us all the reams of misinformation and argumentation, and the scandalous behavior of the anti-hereditarians would, I hope, have been forestalled.

Your present exposure of it, however, is devastating and marvelously accomplished in terms of its penetrating scholarship…I will exert whatever influence I may have to get it reviewed in the most appropriate journals…

Please let me know if your book is definitely “in press” with Cambridge and the likely publication date. I would like to make reference to it in the book I am presently writing…

The thread generates two replies, in which the last user responds: “Wow! What a treasure. The secular equivalent of Luke 7:9, when Jesus marvels at the faith of the Roman centurion, ‘Not even in Israel have I seen such faith.’” The comparison and invocation of Luke 7:9 is particularly telling, in that it positions Jensen as someone with certain unique capabilities to identify belief and adherence. It is this capacity to dole out recognition based on adherence that makes Jensen an authority to be revered, and the reception of such recognition as something to be cherished – “Wow! What a treasure.”

To sum up, the example puts into stark relief how charismatic authority begets charismatic community. The stories that allow acolytes to reinterpret their state of embattlement do not simply embody a discursive logic, but they also reflect a practical logic, in which actors try to access the cachet that comes from charismatic recognition or work to keep such cachet meaningful. Thus, as we detail in the remainder of the section, the cultivation and maintenance of such bonds spreads charismatic authority across time and space, through the efforts of differently placed acolytes.

### Part 2: the charismatic aristocracy

Devotional comportment varies according to access to recognition. Some colleagues and admirers have experienced more sustained interaction with Jensen and therefore can detail more rich encounters with him. Due to this dynamic, there is a cadre of individuals who come to occupy positions similar to what Weber and others have elaborated as the “charismatic aristocracy,” a “select group of adherents who are united by discipleship and loyalty and chosen according to personal charismatic qualification” (Weber, 1978:1119). In more recent writing, Joosse (2017:338) clarifies that what makes this group noteworthy is not that its members have been explicitly or formally selected, but rather that they are “marked by an excellence in their ability to comport themselves as exemplary charismatic followers – followers who are exquisitely qualified to perform roles as valiantly subservient partners in the charismatic interaction.”

Among Jensen’s followers, such a structure comes into being as other similarly placed players give deference to him. One educational psychologist and psychometrician, for instance, describes the negative reception that he and co-authors experienced in the early 1970s, upon conducting studies that concluded that college entrance exams for both blacks and whites alike predicted college performance (Stanley, 1987:7–8):

I was attacked verbally for an hour by the black half of the audience at a private conference, while the other half (whites) remained silent. My work was often assailed in print also. It was considered de facto racist even to investigate the issue…

Yet, when it comes to describing Jensen, this author not only places Jensen at the top of the pantheon but even seems to minimize his experiences:

From my personal experiences I know a *little* about the type of persecution he has undergone, enough to marvel at his resilience, persistence, and patient attitude toward his critics. Any rhinoceros should be delighted to trade skins with him; most of us are far too thin-skinned for more than hit-and-run tactics.

In accounts like these, these colleagues go out of their way to signal that they are not equals with Jensen. To fail to do so would “suffuse” the focus, such that it would “attenuate, if not contradict,” a key distinction “at the heart of the charismatic appeal, namely, the leader’s status as being ‘set apart’” (Joosse, 2017:337).

Members of the charismatic aristocracy help fulfill two key roles. First, they bring an ability to claim that they or others have been *witness* to Jensen’s extraordinary qualities. Typically, these claims focus on how these followers witnessed Jensen endure abuse at public events, respond in a calm, blasé manner, and proceed undeterred. In some cases, though, these stories fixate on Jensen’s peculiar, missionary zeal. One colleague recounts a visit Jensen made to his campus (Detterman, 1998:178):

After the talk ended…Jensen slowly made his way around the room working toward the Communist Party members who were bunched in a corner… They began asking him questions about intelligence which he enthusiastically answered. The conversation went on for some time. The rest of the audience drifted away and the caterers began cleaning up. Jensen carried on enthusiastically and, at least in my opinion, his opponents were loosing [sic] badly. Looking for a way out, the Communist Party members slowly began backing towards the door. But Jensen was just getting started and for every step backward they took toward the door, he took one forward both figuratively and literally. Feeling a bit sorry for them by this time, I told Dr. Jensen that we had to leave for dinner. Taking the opportunity, the representatives of the Communist Party bolted for the door and began walking east on Euclid Avenue. You could see that Jensen was disappointed to loose [sic] his sparring partners.

Accounts like these are littered throughout both the academic and non-academic coverage of Jensen (Nyborg, 2003; Wainer & Robinson, 2009). Through such accounts, Jensen’s chorus of followers contribute to the stagecraft that facilitates his charismatic reputation by creating forms of scholarly folklore. This is a non-trivial practice, as previous writings on charisma have pointed out that charismatic figures can be unaware that followers are encircling them or lack the confidence to assume such leadership (Joosse, 2017:338). In Jensen’s case, perhaps it is as his close colleagues say that he lacked an interest in the limelight, and that his public engagement was only a matter of wanting to advance his findings as any scholar might (Arden, 2003; Wainer & Robinson, 2009). In any case, the chorus of followers buttress Jensen’s charismatic aura by “cloaking” him in “heroic myths,” popularizing such myths for wider audiences, and minimizing the effects of Jensen’s role ambivalence (Joosse, 2012:182–83).

Second, members of the charismatic aristocracy also model devotional comportment to wider publics. In academic communities, the modeling function of the charismatic aristocracy is perhaps not as explicit as it can be in religious communities, where followers sometimes literally show others how to be a devotee (Joosse, 2017:345). However, members of Jensen’s charismatic aristocracy certainly lay signals to encourage aspiring followers. In this manner, they play the role of a hype man or hype woman, as is clearly the case with one intelligence researcher, who is often viewed as a close follower of Jensen (Gottfredson, 1998:297): “For nearly twenty years Jensen would labor under the presumption that his was a minority view in the field of intelligence. And this was despite his knowing that there were many ‘closet Jensenists.’”

Such hyping efforts are not always negative in tone like this. Elsewhere this same researcher, in between such castigations, offers a hopeful overture. When describing the role that Jensen and his colleagues have played in her professional path, she explains (Wainer & Robinson, 2009:421):

They are interesting, independent, and resolute, and they are models of scientific integrity. I have always strived to be like them. I never had much homework in high school, so I would come home and watch old movies on TV. Many were about heroism during WWII, which was still rather recent history. I always asked myself whether I would have done what they did. I hoped I could. It has been like that, watching these scholars over the years, and I hope I have inspired others like they inspired me.

If the coin of the realm in charismatic communities is recognition from the leader, then part of the work that the charismatic aristocracy does is convey that one need not be within close proximity to the charismatic leader in order to cash in. Without such encouragement, aspiring followers would have little incentive to be more than a “closet Jensenist,” given the tales of beleaguerment that otherwise accompany Jensen folklore.

In short, the charismatic aristocracy helps proselytize that the affective state of mind that one gains from revering Jensen can be had by doing “the good work” elsewhere and providing illustrations of what such work looks like. In this regard, the charismatic aristocracy sets in motion the distribution of charismatic devotion across time and space. We explore more distant varieties of these efforts next.

### Part 3: remembrance practices, or how acolytes memorialize the lessons of the charismatic leader

With Jensen’s retirement and eventual passing, charismatic authority faces practical challenges. First, for younger admirers, would-be colleagues, and recent entrants into the world of race and intelligence research, the possibility of interacting with and receiving recognition from Jensen becomes less of a possibility. These individuals are limited in their ability to partake in firsthand storytelling. To some degree, this also becomes a challenge for those within the charismatic aristocracy as well, as no new material is generated for narrating and witnessing Jensen’s charisma. Second, the “spirals of excitement” that feed charismatic authority risk becoming attenuated. As others (Joosse, 2017; Reed, 2013) have described, in order for a charismatic community to continue to thrive, drama and emotional excitement need to accompany the charismatic performance and maintain the sense that the need for charismatic guidance is urgent. When Jensen, the practicing scientist or living individual, is no longer subjected to criticism, the sense of excitement risks becoming a thing of the increasingly distant past. Due to these practical problems, as time passes on, devotional comportment in all likelihood takes different kinds of shapes for those who wish to partake in the public veneration of Jensen.

In this section, we introduce one such kind of devotional comportment – the remembrance practice – and then illustrate in the remaining sections two styles of charismatic veneration that flow from this form, as acolytes recall Jensen’s legacy and recontextualize him in two different ways for the needs of the present. Remembrance practices consist of narratives or archival activities that keep the folklore surrounding Jensen alive. We see this in the way that present-day admirers collect and recirculate firsthand accounts of how Jensen was yelled at by protestors or had to cancel speaking engagements. For instance, users will mine the contents of memoirs written by Jensen’s colleagues, who witnessed such events occur to Jensen, and then screenshot or re-post such passages to Twitter or other online venues. In sum, these activities allow participants to repeat the tales of Jensen’s battles with his detractors. In this manner, some users try to elongate the spirals of excitement that accompanied Jensen’s career by reliving the drama of his time, and they practice devotion by becoming archivists and digital proselytizers of Jensen folklore.

At other times, however, remembrance practices take a related but different turn, as acolytes remember Jensen by re-situating and re-framing him in terms of the crises of the present rather than the past. In these accounts, Jensen is not just remembered as someone who experienced backlash during his career, but is also positioned as someone who experienced “an early manifestation of cancel culture” as one blog post puts it^14^. One academic psychologist – who calls Jensen his “intellectual hero” – invokes Jensen’s life and career in this manner in the introduction to a thread devoted to academic freedom^15^:

“It’s useful to remember what happened to people such as Art Jensen, Hans Eysenck, William Shockley, and J.P. Rushton *before* the supposed rise in social justice ideologies…Jensen had his tired [sic] slashed and was escorted around campus by bodyguards, for example…There is still a surreal public/private gap for many respected professionals, i.e. these professionals say many things privately that they would never say publicly because they are legitimately afraid of backlash and job security.”

In some ways, members of the charismatic aristocracy prime other present-day acolytes for this practice. For instance, one white nationalist journalist who corresponded with Jensen for decades describes the reaction to Jensen as “the start of a Reign of Terror now ten times longer, and vastly more serious, than the much-mythologized McCarthy Era^16^.”

In summary, what we begin to see here is that remembrance practices further develop the Janus-faced aspect of charisma. On the one hand, the constellation of ideas that uphold charisma look towards the persecuted figure, producing scripts that venerate them and imbue them with legitimacy. On the other hand, such scripts look towards the figure’s detractors, producing scripts that construct them as a mob or members of an illiberal, idea-censoring force. Second, by stitching these Janus-faced elements together, remembrance practices provide a mobile form of charismatic veneration. As we see here, they do not offer a direct testimony of having witnessed Jensen’s prowess, but they offer folkloric tales of his extraordinary capabilities as a public figure, any of which can be deployed in debates about suppression of ideas, comments about what “the mob” has done, and discussions about whether a possible path forward exists. Altogether, this is one of the ways that charismatic reverence receives a kind of afterlife, as folk tales about a persecuted figure become something that actors can arm themselves with and use as an interpretive resource in debates about suppression of science in the public sphere. The following sections discuss two such uses.

### Part 4: repurposing the legacy to construct an icon of civility

Sometimes actors invoke features of Jensen’s charisma as a boundary-drawing resource to demarcate what constitutes civil or incivil debate in the public sphere. In many cases, this kind of boundary-drawing is mundane, functioning as a sense-making tool that draws on Jensen’s life. In one Twitter thread, for instance, a user responds to a thread about an essay concerned with free speech by proferring^17^: “People are always against some sort of speech there never was nor never will be a golden age of free speech. Today they are against ‘racist’ speech and going back to probably the late 60s see Arthur Jensen, Hans Eysenck…In the 40s/50s it was communism.”

At other times, this boundary-drawing is polemical, as one prolific, anonymous hereditarian demonstrates in a mini-essay re-posted on a far-right news site, describing the risks that “the activist left” poses to scientific debate^18^:

“The desperation of the left, evidenced in tactics such as its endless smear campaigns against honorable and respected scientists like Arthur Jensen, suggests that it quietly (and perhaps even subconsciously) suspects that the worst is true. Otherwise, why would it so aggressively fight against the idea of funding for rigorous scientific research which should, to their way of thinking, ultimately produce the promised egalitarian result?”

When operating within this polemical mode, this boundary-drawing can indeed become less diagnostic and more prescriptive, as acolytes demarcate good/bad speech by using elements of Jensen’s life to describe virtuous speech. The practice that most epitomizes this pattern is the way that acolytes invoke Jensen’s scholarly conversations with James Flynn, an intelligence researcher who has become positioned as a “leftist” or “environmentalist.” While some admirers do discuss the specifics of what Jensen and Flynn disagreed upon, and what points each tended to focus upon, often references to this relationship simply focus on the collegial correspondence between Jensen and Flynn. In this manner, Jensen’s conversations with Flynn become evidence of a certain kind of miracle, which is that discussion about the problem of race differences can indeed engender civil dialogue. This collegial dynamic may have initially been observable to close interlocutors, but with time it has become a piece of folklore in its own right. Users on Twitter, for instance, share screenshots of the opening to a book written by James Flynn, in which he dedicates the book to Jensen^19^. Elsewhere, hereditarians implore their detractors on Twitter to “study” the discussion between Jensen and Flynn, as one white nationalist journalist urges, in the middle of a back and forth between an anti-racist biologist geneticist and a hereditarian psychologist^20^: “The interchanges between James Flynn and Arthur Jensen should be taught in philosophy of science courses as a model for scientist behavior.” By recalling Jensen’s conversations with Flynn, admirers seek to typify what they think is the model of civility that they ought to strive for as scientists.

To put a further point on what qualifies as ideal speech, acolytes often mention Jensen’s engagement with Flynn to challenge the charge of racism that comes up in relation to Jensen and intelligence research. As the argument goes, Flynn’s engagement with Jensen not only establishes that talk about racism ought to be tabled, but it proves that liberals or environmentalists are uncharitable and too quick to bring up the charge. This way of appropriating Jensen’s and Flynn’s dialogues comes out in the following exchange between an anti-racist evolutionary biologist and a hereditarian psychologist^21^:

itsbirdemic: He was a racist and on racial differences he didn’t know what he was talking about. It was enough to be an entire chapter in his tributed book edited by fucking Nyborg.

EPoe187: You use that word very casually. People who actually know the literature, say James Flynn, praised Jensen profusely and claimed he was a gentleman without a scintilla of racial bias. Possibly, you should actually read the literature first.

Thus, by recounting tales of the once civil Jensen-Flynn exchanges, acolytes can momentarily respond to labels of racism, marking them as unwarranted or as a form of talk that in its own right is uncivil.

In brief, one of the ways that charisma lives on even after death is through a particular manner by which acolytes “spread the gospel.” Storytelling about the leader’s prowess not only becomes a means of breathing life into the charismatic mythos, but it also provides a maneuver by which acolytes can attempt to engage others in public conversations, to redefine which of the conversational interlocutors is an uncivil member of the public sphere. In particular, acolytes can use detractors’ inabilities to appreciate the miracle at the heart of the charismatic leader’s prowess as a way of signaling who is uncharitable, and recharacterizing accusations of extremism as the real kind of extremism.

### Part 5: repurposing the legacy to construct a cautionary tale

At other times, acolytes leverage memories of Jensen’s charismatic performance to proffer a quite different and even seemingly contradictory lesson about civility in public discussions. In this mode, acolytes question whether the extraordinary civility that Jensen embodied – and to which they signal deference – is the right path forward for them and others. Seeds of this ambivalence appear in the quotes of some of Jensen’s closest colleagues, as some remark that he was simultaneously saintly and ill-equipped for the limelight. Charles Murray, for one, has described Jensen in such terms in multiple places. At the same time that he tells his social media followers “no one deserved vilification so little, and put up with so much of it so gracefully as Art Jensen,” he also tells journalists (Woo, 2012b):

“Although he had this reputation as a very controversial figure, he was actually a pure academic and almost a naive one…He was…devoted to analysis and kind of obtuse about the reaction he would provoke with the findings he came out with.”

One white nationalist journalist – the same one who said Jensen bore the brunt of a new era of McCarthyism – recasts this property of Jensen’s in more pointed terms, remarking that Jensen’s attempts to convey to audiences that he had some progressive leanings “did Jensen no good: for his conclusion that genetics accounts for 50 percent of racial differences in intelligence, he was treated as a pariah^22^.” In sum, amidst all the fawning, there is also a sense at times among acolytes that Jensen may have been “too good for his own good.”

Without a doubt, some version of this ambivalence is likely in many charismatic communities, as acolytes question whether they have the wherewithal to be as extraordinary as their figurehead or try to rationalize why they can never be as remarkable. Importantly, in the context of Jensen and his followers, this ambivalence itself becomes an interpretive resource with which acolytes make sense of public debate, and in many cases results in the verdict that they should not be civil. We see this determination in action with one active racial hereditarian user, who in a Twitter debate admonishes one of his allies for trying to play some version of respectability politics when questioned by another user^23^:

You should stop letting her play the dissociation game with you. You cannot win by bad mouthing Pioneer Fund or Richard Spencer, etc. They will always hate you because they hate reality of group differences, no matter your personal virtues.

Arthur Jensen was basically a saint, it did not help him. Phil Rushton, not so much, and he got the same treatment. Hans Eysenck fled Nazis – still gets called Nazi, etc. Charles Murray is a very nice person, and what good did it do him? Block malicious users (her) and move on.

This suggestion turns into a more direct statement of principle later, when the same user reviews an episode of a podcast on intelligence. In one portion of the episode, two academic psychologists discuss Jensen’s career controversies, and one of them cautions that intelligence is too complex and emotionally-charged of an issue for debate on social media. It’s in regard to this portion that this hereditarian user urges^24^:

…*I reject this false standard where hereditarians must be flawless, disinterested people whereas environmentalists can be openly ANTIFA-supporting Marxists, who wish death upon their opponents.* I cannot stand the lying academics and the double standards. The nice intelligence researchers have themselves to blame for the race scientists being odd characters. They themselves refuse to get their hands dirty with the data. They prefer these high-horse takes about the bad character of the race scientists. [emphasis in original]

To be sure, this kind of ownership of coarse online behavior demonstrated above is rare; other scholars have commented that one of the defining features of online incivility – e.g. trolling, shitposting – is plausible deniability, as well as posturing in which users describe others as the ones who are over-reaching or over-interpreting one’s behavior (Fielitz and Thurston, 2019). Similarly, in this regard, such an explicit re-framing of Jensen’s legacy as something that mandates incivility is uncommon. Yet, less explicit shades of this practice shine through among other Jensen acolytes. One illustration of this comes in a book review written by a far-right journalist and lay racial hereditarian – Steve Sailer – who expresses intense admiration for Jensen and encourages others to model themselves on the afore-mentioned Jensen-Flynn dialogue. In his review of *Superior*^25^, a recently published popular science book exploring recent developments in race science, Sailer begins by sarcastically declaring: “In Angela Saini’s book *Superior*…I am cast as a villain, along with Francis Galton, Arthur Jensen, James D. Watson, Morrisey, David Reich, and even Albert Einstein.” He then adds “the lineup makes me feel like the batboy on the 1927 Yankees: honored just to be on the same field.” It’s unclear how impactful this “honor” is though: while part of Sailer’s review involves disputing events in which he is involved, or predictably castigating Saini as a science denier, other elements involve a less-than-Jensenesque form of pushback. In part of the review, Sailer describes Saini as someone “[who] takes everything personally in that girly style that predominates in 2019,” as well as “a loyal Indian racialist…[who] has an obsession with finding sciencey-sounding arguments that her people have just as much right to move to England as the native English have to live there.”

In summary, these moments illustrate that in the same statement acolytes can breathe life into the mythos surrounding a charismatic figure while simultaneously deciding to be fairly un-charismatic. When made most explicit, the sentiment that accompanies these moments here suggests that Jensen is a person to be admired but also someone whose virtuous behavior yielded little personally for him and the broader research enterprise he stands for generally. In others, it results in a more inchoate sense that his admirable life need not be the guide for all who choose to admire him – there may be honor in paying tribute to and being lumped in with Jensen, but also little glory in meting out one’s public engagement like the charismatic leader himself did. In either case, this is one of the ways that acolytes interpret Jensen’s legacy to make sense of how to navigate public dialogue, by surmising from Jensen’s career that civility is a foreclosed process.

In sum, this analysis points to one of the ways in which charisma is an unstable form of authority – not because it risks being usurped and incorporated piecemeal by bureaucracies and other forms of rational authority, as most theorists usually point to (Dow, 1978; Weber, 1978). Rather, it is because the same interpretations that result in the legitimation of charismatic figures can also be used to generate scripts that undermine those figures, resulting in, first, a kind of devotional ambivalence and, second, behavior on the part of acolytes in which they seem like as much a part of the rabble that they excoriate elsewhere.

## Discussion

### Science and charisma

The case of Arthur Jensen and charisma provides some instructive lessons about how science becomes influential and lives on in the public. To begin, many sociologists of science commonly think about professional validation as crucial to understanding how scientists become influential to one another and the public. In contrast, our case illustrates how scientists can forge alternate routes for influence outside of such procedural and gatekeeping channels, by creating a charismatic community that enrolls supporters with concerns about propriety. In this mode, “enrolling the public” means something different than often the case in science studies; rather than a sociotechnical arrangement at the center, for which civil society actors must be found to help translate or co-produce stability and formation (Eyal, 2013; Jasanoff, 2004), we see here a situation in which scientists are trying to make normative a certain model of scientific discourse, and in doing so activating a public drama around which lay experts and science hobbyists can play a role.

By centering the role of charisma, our article helps expand existing accounts of charisma, both generally and specifically with science. Generally speaking, charisma theorizing is often fleshed out in connection to “the most vivid examples of what Weber called ‘pure’ charisma,” which “seem to apply most consistently to religious leadership” (Joosse, 2017:351). Our article responds to Joosse’s call to see the development of charisma in a wider range of fields with more attention to field-specific structures. Specifically in regard to science, one of the field dynamics key to charisma’s genesis appears to be the role of an ongoing professional conflict, which not only divides sides but positions one set of players as marginal and unorthodox. Such a professional conflict also sets in place the conditions for the second key field dynamic, which is beleaguered players’ appetite for a seemingly legitimate player, particularly one whose contributions are belittled and whose rebuttals demonstrate remarkable equanimity. In relation to these dynamics, embattled practitioners and admirers begin responding to scientific debates by drawing on the symbolic resources that the seemingly legitimate player’s performance offers.

Our case suggests that this public drama and the charismatic grouping at its center may be highly reliant on what Collins and Pinch (1979) term “contingent forums.” In contrast to constitutive forums, like “learned journals” and “formal conference settings,” contingent forums involve: para-academic publications, like festschrifts or editorial sections of journals; popular venues, like news interviews, blogs, and social media posts; as well as other spaces, in which “discussion,” “gossip,” “fund raising,” and “publicity seeking” thrive. As we see here, Jensen’s colleagues have taken the occasion to produce three edited volumes in his honor (i.e. festschrifts). These are the venues in which first-person accounts of experiencing his extraordinary presence flourish, whether it be stories about witnessing him endure public haranguing, testimonies about his principled but generous demeanor, or reflections about the influence he has had on one’s career. Additionally, his distant as well as lay admirers have found space in white nationalist publications, tweets, and blog posts, among other venues, to amplify his stature. These are spaces in which Jensen’s charisma takes on the shape of a myth – more often than not, a second-hand recollection that speaks to some miraculous individual who one would be lucky to meet. In some ways, this aspect of contingent forums speaks to the enduring power of gatekeeping channels like peer review and their ability to limit the circulation of perspectives that cannot pass the muster of professional validation. On the other hand, a charismatic group’s ability to advocate and network within contingent forums illustrates the ability of embattled players to reconfigure the terrain of science, by creating a public that spans disciplines, academic/non-academic boundaries, and public communication venues.

Fundamentally, these broad points allow us to rethink some of the conventional ways that scholars think about charisma’s connection with the institution of science. First, our case underscores the need to think about charisma as much of a multi-sited, relational phenomenon as it is an attribute. While most studies of charisma acknowledge that there is a leader/follower or influencer/influenced relationship at play, many of these studies ultimately locate the genesis of this relationship within an indescribable marvelousness (e.g. attribute) that the charismatic figure allegedly possesses. In the science studies literature, this particular reading has also fostered a cadre of studies that describe technoscientific objects as charismatic figures, which conjure feelings of wonderment about what science, technological development, or the natural world is capable of (Bear, 2013; Lorimer, 2007; Mathews, 2014). A closer look at the dynamics of charisma provided by our article helps clarify that feelings of wonderment alone are perhaps an insufficient basis for invoking the term charisma. As our case indicates, Jensen’s charisma emerged as much from the way a network of actors strove to present him as extraordinary – a key point, given that he also had a reputation for not possessing the attribute of “charisma” as colloquially understood. Thus, the sense of wonderment that people quickly associate with the term is inextricable from a *structure* of authority, and must be analyzed as such. While such inscrutable wonderment can be significant and noteworthy, our study suggests this attribute: a) may be better understood by a different Weberian lexicon, relating to enchantment or re-enchantment within the rationalized sphere of science (Stoliarova, 2021); or b) needs to be further situated as the product of a community, organized around its mythical preservation, in order to be more compellingly described as a case of charisma.

Second, these considerations help us re-appraise issues related to meaning and science and inform when to turn to charisma as a form of analysis. Typically, scholars posit that once individuals discern that institutions of rational authority cannot answer broader questions pertaining to meaning, charisma has the opportunity to rear its head (Thorpe & Shapin, 2000; Weber, 1978). In some cases, this is an issue related to scientists’ implicit awareness that science is a condition in which more knowledge begets more questions (Thorpe & Shapin, 2000). In others, it’s an issue related to scientists’ practical problem that technical knowledge does not necessarily provide clear-cut templates for how to organize research and development units and coordinate workflows (Lengwiler, 2006). In either case, charisma offers directive affordances, functioning as a meaning-laden stopgap to goose decision-making along. Though the broad contours of this interpretation are not disqualified by our case, we posit that the more fine-tuned understanding of the problem here is that charisma may become actionable when individuals (i.e., scientists and science enthusiasts) come to view the institutions of rational authority as corrupted rather than devoid of meaning.

With this dynamic in mind, future research may consider that charisma might not just overlap with conservative or conservative-leaning forces operating within science. While we have used the race and intelligence debates as a case to illustrate, it is important to remind ourselves of the possibility that charisma can retain “value neutrality,” as Weber puts it, at the level of analysis and application. For instance, those with left- or liberal-leanings in science – such as scholars challenging the under-representation of women and racial minorities – also often speak about beleaguerment and embattlement (Charles and Thébaud, 2018). Future research may seek to study whether those scholars in some way activate charisma within their circles.

### Race science and charisma

This brings us to some reflections on the problem of race science. Typically, scholars have treated this subject as a kind of fringe science. They acknowledge that it can be impactful, but focus on either: 1) the gap between the underlying scientific merits and the public discussion it engenders or 2) the social harm that such fringe scientific representations of race can set in motion. Ultimately, we are left with an underdeveloped sense of why this research thrives, other than that it provides scientific-seeming rationales for racist ideologues. The study of how others receive Jensen and continue to propagate his mythos illustrates that such research may thrive in part because the social context of such research involves actors capitalizing on well-understood tropes of incivility, persecution, and martyrdom. These kinds of claims and presentations conjure up support both inside and outside academia. Just as importantly, they help such research gain legitimacy in a way that is unrelated to its scientific merit or the underlying informational content, and these processes help convert questions about the social implications of such research into charges of incivility.

In this manner, this paper builds on previous work by one of us which explores how “images of strength” are fundamental to the lifeline of race and intelligence research (Panofsky, 2018). Such research illustrates that an “arm’s length embrace” of scientific racism helps scientists cultivate professional authority, as such posturing provides the ability to project strength, autonomy, and assurance. Thus, this earlier work also offers a vantage point into how scientific legitimacy (and thus authority) can be built without increasing the *credibility* of scientific contents. In total, the present paper and this previous research help re-appraise a particular aspect of the ongoing race and IQ debate, which as some behavior geneticists have pointed is its longstanding empirical/scientific stagnation – “that there has been no new direct evidence” pertaining to the genetic hypothesis for black-white differences in IQ for decades now (Nisbett et al., 2012). The significant role that non-evidentiary sources of authority play in the field’s legitimacy suggest that such stagnation need not be read as a bug, but rather very much a potential feature of the field.

The focus on charismatic organization points to some potentially vexing conclusions about the public life of race and intelligence research. In large part, the hagiography of this topic has been marked by a focus on either scholarly research or the impact of certain high-profile publications – such as Jensen’s *HER* article or books like *The Bell Curve*. Our research departs somewhat, as it reveals a different manner through which this topic gains public longevity. What we discover is that a more motley mix of players – not all of whom are established academics – are capable of providing this topic with publicity in a way that is less dependent on – though still tied up with – academic publications that create noise or receive coverage in *The New Republic*. Moreover, these actors need not wait for academic and media elites to orchestrate controversy and provide the affective resources that renew involvement among the committed. Instead, they have at their disposal more mundane tools – e.g. folkloric rituals, social media activity – to keep alive the sense of excitement that animates this topic. Altogether, for those interested in challenging biodeterminist views, this reality presents dilemmas. For one, arming oneself with facts and figures may be of limited value, since much of what binds this racial hereditarian community together is of a non-evidentiary form. In short, one will be confronting actors vested in a relational project, separate from its status as a questionable scientific perspective or questionable social perspective. Additionally, engagement in the form of arguments and rebuttals may also add to the sense of excitement upon which charismatic veneration thrives, providing resources that may reaffirm the particular symbolic and affective modes through which this research gains public traction.

These insights are worth grappling with in the present context, because race scientists are not the only ones trading in on such cultural structures. There is a burgeoning cadre of scholars who are creating similar projects under the banner of “heterodox science” or “contrarian science” (Aupers and de Wildt, 2021; Parks, 2020). These efforts are structured similarly, in that they exist as loose academic groupings; sometimes they are anchored by conferences or nascent professional organizations, but they are just as much held together through social media interactions, op-eds in new media, and other internet-based platforms – or, in other words, through contingent forums. More to the point, members of these scholarly communities bind themselves together through stories about persecution or the sciences becoming corrupt and intolerant. To be sure, not all who advocate for the creation of these communities are involved in scientific research that feeds prejudicial viewpoints or has been deemed dubious by peers and publications. However, these kinds of communities and the narratives they circulate provide a kind of refuge for such scholars, by providing interpretive resources that: 1) reframe embattlement as evidence of scholarly incivility and 2) confer legitimacy to a scholar based on his or her public performance and engagement with critics, rather than the contributions that she or he has made to a field of research. Altogether, the specific case in this article helps us begin to understand some of the kinds of intellectual entrepreneurship that we see behind the efforts of other aspiring scientific public intellectuals in today’s public sphere.

## Footnotes

1 Tweet available at (https://web.archive.org/web/20201214171043/https://twitter.com/rjhaier/status/1338530546373181448)

2 Tweets available at (https://web.archive.org/web/20200731233628/https://twitter.com/EPoe187/status/1289344133849522176) and (https://web.archive.org/web/20210114182823/https://twitter.com/KirkegaardEmil/status/1349785319948906496)

3 Example at (https://web.archive.org/web/20210413063154/https://russellwarne.com/2020/12/05/notes-on-videos-of-arthur-jensen-defending-his-work/)

4 See the journal article titled “Dodging Darwin” published in 2020 in *Personality and Individual Differences* for an example. See the following punditry pieces too: (https://web.archive.org/web/20171201135552/http://quillette.com/2017/11/29/politics-science-scientists-might-not-say-evidence-supports/); (https://web.archive.org/web/20160811060408/https://quillette.com/2016/08/09/on-the-reality-of-race-the-abhorrence-of-racism-part-ii-human-biodiversity-its-implications/); (https://web.archive.org/web/20170207210105/https://quillette.com/2017/02/06/dealing-with-the-reality-that-not-everyone-can-succeed/)

5 See Arthur Jensen’s article he co-authored with J. Philippe Rushton in 2005 in *Psychological Science*, which near the end of his life summarized – to his mind – the most compelling evidence.

6 See Miele (2002:87–88) for one example of this.

7 See Jensen (1974) for these biographical details.

8 See Jensen (1967) for an example.

9 See any of the three festschrifts (Detterman, 1998; Modgil & Modgil, 1987; Nyborg, 2003) published in honor of Jensen for easily accessible accounts of colleagues describing Jensen in such terms, which are otherwise recited in a range of places across scholarly writings, popular press accounts, and social media.

10 For very easily accessible accounts of such supportive and skeptical observers, see the letters to the editor in a 1970 issue (volume 26, issue 7) of the *Bulletin of Atomic Scientists* that was published after a dialogue in the same journal between Arthur Jensen and Richard Lewontin.

11 “Campus Totalitarians,” *The New York Times*, p. 46, published May 20, 1969.

12 See Kaufman (2020) for interview with the quoted psychologist.

13 Tweet available at (https://web.archive.org/web/20191129132920/https://twitter.com/NSesardic/status/1200401598603780098).

14 Available at (https://web.archive.org/web/20220117195018/https://orthosphere.wordpress.com/2022/01/17/iq-determinism/); see also (https://web.archive.org/web/20220307230156/https://edwest.substack.com/p/eo-wilson-and-the-birth-of-cancel?s=r)

15 Available at (https://web.archive.org/web/20220308185555/https://twitter.com/EPoe187/status/1146034408241160192)

16 From a VDare.com article by Kevin Lamb, available at (https://web.archive.org/web/20121104011342/https://vdare.com/articles/arthur-r-jensen-s-courageous-career-choice)

17 Tweet available at (https://web.archive.org/web/20220307215100/https://twitter.com/Hereditarian98/status/988933773185073153?s=20&t=lIwWAp8QmWUVQZJ1WtIfMQ)

18 Available at (https://web.archive.org/web/20190122085212/https://www.unz.com/isteve/is-science-racist/)

19 Available at (https://web.archive.org/web/20200517201452/https://twitter.com/TearsOfGypsy/status/1262112499635089408)

20 Available at (https://web.archive.org/web/20220307232919/https://twitter.com/Steve_Sailer/status/1153200611153682432?s=20&t=X-0A_Eo54pnXBqAhZpKCug)

21 Available at (https://web.archive.org/web/20220307233433/https://twitter.com/thebirdmaniac/status/1152976191336472576)

22 Available at (https://web.archive.org/web/20121104011342/https://vdare.com/articles/arthur-r-jensens-courageous-career-choice)

23 The following provides some context for this tweet. The Pioneer Fund is a private foundation created shortly after World War 2, and throughout its history it has funded the research of academics who seek to study differences among races and explain them as objective and biologically-rooted phenomena. Richard Spencer is an American neo-Nazi and white nationalist. Tweet available at: (https://web.archive.org/web/20190608234740/https://twitter.com/KirkegaardEmil/status/1137506220493987841)

24
https://web.archive.org/web/20220308000310/https://emilkirkegaard.dk/en/2020/06/richard-haier-on-the-nature-of-human-intelligence-the-psychology-podcast-scott-barry-kaufman/

25 Available at (https://web.archive.org/web/20210416035407/https://www.takimag.com/article/arguing-against-reality/)
